# Application of leaf multispectral analyzer in comparison to hyperspectral device to assess the diversity of spectral reflectance indices in wheat genotypes

**DOI:** 10.1515/biol-2022-0989

**Published:** 2024-11-16

**Authors:** Andrej Filacek, Marek Zivcak, Maria Barboricova, Marek Kovar, Andrej Halabuk, Katarina Gerhatova, Xinghong Yang, Pavol Hauptvogel, Marian Brestic

**Affiliations:** Institute of Plant and Environmental Sciences, Slovak University of Agriculture, Nitra, Slovak Republic; Institute of Landscape Ecology, Slovak Academy of Sciences, 814 99, Bratislava, Slovak Republic; College of Life Science, National Key Laboratory of Wheat Improvement, Shandong Key Laboratory of Crop Biology, Shandong Agricultural University, Taian, 271018, China; National Agricultural and Food Centre, Research Institute of Plant Production, 921 68, Piešťany, Slovak Republic

**Keywords:** non-invasive methods, leaf spectrometer, *Triticum*, genetic resources, vegetation indices, crop phenotyping

## Abstract

Multispectral devices have a huge potential to be utilized in biological, ecological, and agricultural studies, providing valuable information on plant structure and chemical composition. The aim of the study was to assess the reliability and sensitivity of the affordable leaf spectrometer PolyPen (PP) in comparison with the highly sensitive analytical device FieldSpec-4. Measurements at the leaf level were realized on a collection of 24 diverse field-grown wheat (*Triticum* sp. L.) genotypes in several growth phases during the regular growing season, focusing on whole spectral curves and a set of 41 spectral reflectance indices. As expected, the sensitive analytical device showed a higher capacity to capture genotypic variability and the ability to distinguish seasonal changes compared to a low-cost multispectral device. Nevertheless, the analysis of the data provided by low-cost sensors provided a group of parameters with good sensitivity, including reasonable correlations between the records of the two devices (*r* > 0.80). Based on the large obtained datasets, we can conclude that the application of a low-cost PP leaf spectrometer in plant and crop studies can be efficient, but the selection of parameters is crucial. Thus, the present study provides valuable information for users of affordable leaf spectrometers in fundamental and applied plant science.

## 1 Introduction

Wheat is one of the most important cereal crops, and it gets wide attention worldwide [1]. It is essential to increase crop production efficiency through the creation of optimized smart farming practices [2] and various phenotyping approaches [3,4,5] to alleviate worries about future wheat shortages. Field phenotyping is a widely used approach for identifying well-adapted germplasm to specific environments and understanding their basic biology [6]. In recent years, new tools and techniques such as spectral radiometry and other indirect sensing approaches have been created to help in the phenotyping of plants in the field [7]. With respect to other laboratory techniques, spectrometry has the advantage of not being an invasive or damaging technique [8]. Furthermore, a more detailed analysis of the leaf contents is feasible due to the high spectral resolution of spectrometers [9]. Spectral sensors, which can be deployed on the ground, in the air, or in space, work on the basis of reflectance and variations in electromagnetic radiation between 300 and 2,500 nm [10,11]. Low spectral and spatial resolution (10–60 m) is one of the drawbacks of space-born sensors [12,13]. Due to the fact that higher spatial resolution is achieved when the sensor is closer to the object, handheld sensors, sensors installed on vehicles or operated by unmanned aerial vehicles are particularly used in agriculture for their high spatial resolution [14,15].

Sunlight, among other factors, is the most important element in plant photosynthesis, which explains why the majority of research in this field is focused on the spectral characteristics of leaves and canopies [16].

Spectral reflectance analysis is currently a valuable tool in plant ecophysiological research. The light reflected from a leaf is determined by two components: diffusive and specular components [17]. A diffuse portion of reflected light is determined mainly by water content [18], intercellular air spaces [19], the orientation of cell walls in the leaf mesophyll [20], and biochemical components of the leaf [21]. A specular portion of reflected light ignores the internal structure and composition of leaves and is determined by the structural properties of the epidermis and never enters the leaf [22]. Reflectance in the visible band (400–800 nm) is mainly determined by absorption characteristics of the photosynthetic leaf pigments, i.e., chlorophyll a, chlorophyll b, and carotenoids [23]. The major carotenoids of functional chloroplasts of higher plants include beta-carotene, lutein, violaxanthin, and neoxanthin. The frequent but minor carotenoid components consist of xanthophylls, zeaxanthin, and lutein epoxide [24]. The most accurate individual wavebands for determining pigments were found to be 680 nm for chlorophyll a, 635 nm for chlorophyll b, and 470 nm for carotenoids [25]. The anthocyanins are also involved in the absorption of light within the blue-green to orange spectral region (around 450–600 nm) [26] with an absorption maximum of around 529 [21].

Spectral reflectance in the VIS region is relatively low, meaning that the leaf absorbs a larger proportion of incoming light across the spectrum due to the presence of leaf pigments, resulting in less light being reflected. Chlorophyll has the highest absorption in the red and blue spectral areas; its maximum reflectance occurs in the green wavelengths (560 nm), and its maximum absorbance occurs between 660 and 680 nm [27]. Although there are no water bands in the VIS range (400–700 nm), previous studies have found that this region’s wavelengths can still be used to determine a plant’s water status indirectly by observing how drought affects the characteristics of the leaf pigments [28]. The reflectance of UV light from leaves is not expected to be comparable to the commonly measured reflectance of visible light. Large chromatophores and vacuoles, internal scattering at intercellular air spaces, and internal scattering linked to the geometries of palisade cells all have a significant impact on the scattering and transmission of radiation in leaves [29,30,31]. According to Xiao et al. [19], the columnar palisade cells minimize light scattering and enable light to penetrate deeper into a leaf. By contrast, the spherical spongy cells were more effective in scattering light and thus maximized light absorptance for the whole leaf.

The reflectance of the leaf considerably rises on the transition from red to NIR wavelengths, resulting in a distinctive spectral pattern known as the “red edge” [32]. The red edge corresponds to a wavelength, defined mathematically as the inflection point position on the slope connecting the reflectance in the red and the NIR spectral regions [33]. Due to the significant absorption of chlorophyll in the range of 670–680 nm, the absorption can become saturated. Chlorophyll absorption in the near-infrared range is no longer a factor in reflectance above 730 nm [34].

Variations in reflectance and absorptance between stressed and healthy leaves in the 400–500 nm, 670–680 nm, and near-infrared spectra are usually negligible. It has been found that stressed leaves typically have high enough contents of carotenoids and other accessory pigments to maintain equivalent absorption in the 400–500 nm region [35]. Accumulation of carotenoids due to heavy metal stress was detected in *Vicia faba* [36] and *Cicer arietinum* [37]. It has also been observed that shortages in phosphorus and potassium elevate the amount of carotenoid in spinach [38]. Various stress conditions triggered the accumulation of β-carotene in *Dunaliella salina* [39]. The effect of UV-B radiation caused the accumulation of carotenoids in the leaves of *Nicotiana tabacum* L. [40]. Munné-Bosch and Alegre [41] found that during drought stress, the rates of degradation of chlorophylls and β-carotene were comparable; however, the lutein to chlorophyll ratio increased in *Rosmarinus officinalis*.

The precise measurement of pigments, water content, and leaf structural factors provides insightful data on plant stress and biomass yield. More and more parameter-specific indices are created by selecting appropriate band combinations [42]. There have been many narrow-band indexes proposed in the literature [14]. To calculate the vegetation index (VI), mathematical formulas involving two or more spectral bands must be incorporated [43]. Utilizing VIs based on narrowband or hyperspectral data has become more popular as spectroradiometers with the capacity to gather data in very narrow bandwidths (1–10 nm) and continuously over the spectral range have become more widely available [44]. The majority of uses for these narrowband indices are for the analysis of leaf pigment content [45]. For the calculation of these indices, the wavelengths of the red edge region (670–780 nm) in combination with the wavelengths of the VIS band are often used [46]. Examples include the MERIS terrestrial chlorophyll index (MTCI), red-edge position (REP), the Structure Insensitive Pigment Index (SIPI), and the Plant Senescence Reflectance Index (PSRI) [33,35,47,48]. The most often used index for assessing the vigor of vegetation is the normalized difference vegetation index (NDVI), although this does not mean it is always effective [49].

It is commonly accepted to categorize fieldable spectrometers into three categories: transportable (e.g., deployable in the field while mounted in a car), portable in “suitcase” format (weighing more than 4 kg of total equipment weight), and handheld (weighing 1 kg) [50]. Portable spectrometers are designed for field use, unlike benchtop instrumentation, which is typically used only in laboratory settings [34]. Reflectance measurements with handheld spectroscopic equipment for handheld spectroscopy are frequently utilized in agronomic research to take point measurements with multiple replications across larger plots [50].

Various series of non-imaging spectrometers, such as FieldSpec-4 (FS-4) (ASD, USA), are available [25]. However, these devices are too expensive for even smaller farmers to consider buying them. More affordable, portable, and easy-to-use instruments are needed to make hyperspectral technology available to the farming industry. One of the candidates is PolyPen (PP) (PSI, Czech Republic), which is presented as a device convenient for indoor and field applications. The trade-off with such instruments is that their spectrum ranges and resolutions are often lower compared to high-resolution scientific instruments, and hence, the results might not be as robust. On the other hand, the information provided by the low-cost sensors may be sufficient for some applications, depending on the variation of the data and the aims of the assessment. Most examples of successful application of low-cost sensors are limited to studies on nutrient deficiency [51,52], pathogen impact [53], or seasonal impacts [54]. The information on the genotypic variability in spectral reflectance and the ability to record the genotypic differences using the handheld spectrometers are rarely presented, and a valid analysis comparing the spectrometers in a variable set of crop genotypes is almost missing. Considering an urgent request to provide valid, affordable technical tools for assessing genetic resources and breeding populations, our study focused on comparing two sensors analyzing well-established field collections of diverse wheat genotypes during the regular growth season. In addition to comparing the spectral records, we focused on analyses of a set of spectral reflectance indices obtained by a low-cost and standard scientific spectrometer, searching for the parameters with a suitable capacity to recognize the genotypic variance and seasonal changes and reliability demonstrated by correlation analysis.

## 2 Materials and methods

### 2.1 Plant material and growth conditions

Research experiments were carried out in a regular growing season with a field-grown wheat collection (field trials, Gene bank of Slovak Republic at National Agricultural and Food Centre – Research Institute of Plant Production in Piešťany: 48°35′10.8″N; 17°48′36.8″E; 156 ALT). For experimental purposes, 24 morphologically and physiologically diverse wheat genotypes were used, namely: Equinox (GBR), Dattel (FRA), Thesee (FRA), CGN 04265 (EGY), 16/26 (SVN), Magnif 27 (M.G.) (ARG), President Riverain (FRA), Landrace 1-96 (DEU), Rajve (SVN), Sloga (SRB), Japan 1620 (JPN), Zun 4 (CHN), San Pastore (ITA), Kotte (SWE), 2010K11-10 (CHN), GRC 867 (GRC) (*Triticum aestivum* L.), Dusan (SRB) (*Triticum durum* Desf.), AZESVK2009-97 (AZE), Spelt lijn 73 (BEL) (*Triticum spelta* L.), Roter Samtiger Kolbenweizen (DEU), Unmedpur Mummy (EGY), AZESVK2009-90 (GEO), NP 202 (New Pusa) (IND), and AZESVK2009-88 (GEO) (*Triticum turgidum* L.). The seeds were sown by hand directly into the soil in the fall on experimental plots with an area of 1.5 m^2^.

To detect substantial water deficits in field conditions, the crop moisture index, a drought index that takes temperature and precipitation into account to predict the soil water balance, was calculated for the vegetation period [55]. When water deficit in soil was observed, irrigation was initiated out in drip form with an approximate dose of 2 m^−3^ once a week.

### 2.2 Measurements of spectral reflectance

Measurements of spectral reflectance were realized in laboratory conditions. The youngest fully developed leaf from each genotype was directly separated in the field for the measurements; it was put in plastic bags with a quick-lock closure and transported as quickly as possible to the laboratory setting, where it was then put in a beaker with water to prevent dehydration. Before inserting it into the measuring clip of the device, the leaf was taken out of the beaker and gently dried with paper cotton. One plant represented one replicate; the experiments and measurements were performed in 10 replicates (*n* = 10) per genotype. Measurements were taken in the middle of the leaf blade of the adaxial side of each leaf, avoiding the midvein. Due to the fact that we worked with wheat at the Z39-47 stage [56], the leaves were sufficiently developed to cover the entire measurement area of the contact probe (10 mm). Three points of each leaf of the individual genotype were measured, and the average of the three points was calculated.

The spectral reflectance curves were measured using the broadband spectroradiometer ASD FS-4 (ASD Inc., Boulder, CO, USA) with a measuring range of 350–2,500 nm and with a multispectral spectroradiometer PP RP410 (PSI Ltd., Drasov, Czech Republic) where the UV/VIS version of the detector with a measurement range of 380–790 nm was used. Both spectroradiometers were used to analyze single-leaf reflectance. The spectral reflectance was collected from the leaf’s adaxial side. Measurements with ASD FS-4 involved using an ASD contact probe and a leaf clip (ASD Plant Probe; ASD Inc., USA) specifically designed for plant leaves. The ASD contact probe is equipped with a halogen bulb and is capable of taking reflectance measurements with a spot size of 10 mm. To create relative reflectance spectra, the radiance spectra of the leaf reflectance between 350 and 2,500 nm were normalized against a white reference using 99% Spectralon (Spectralon SRS99, Labsphere, North Sutton, US) [42]. To set up the equipment, a reading of the dark current signal, obtained with the fiber optic shutter closed, was also taken. This reading indicated the fraction of the signal originating from within the equipment and was removed from all subsequent measurements. Since the measurements were carried out in laboratory conditions, white and dark reference data were taken prior to the first measurement and subsequently after 10 measurements [57]. To reduce background spectral noise or radiation passed via the leaves, they were placed on a piece of foam rubber which matches the requirements of a low-reflectance standard for laboratory measurements of spectral characteristics [58]. The contact probe features a consistent light source built into the mount for the spectroradiometer optic cable. The light source of the contact probe is positioned at a 23° angle relative to the probe body. At a fixed view zenith angle of 35°, the spectroradiometer optic cable is positioned in the contact probe in the same azimuth about the probe axis as the light source [59]. A fiber optic contact probe was put on a leaf surface, and the entire illumination for the leaf surface came from the contact probe itself.

A leaf contact active spectrometer with a 1 nm resolution, the PP measures wavelengths between 330 and 790 nm. This instrument is already equipped with a non-destructive sample holder with a leaf clip, while the light beam hits the sample at an angle of incidence of 35°. The PP device integrates an internal light source (Xenon incandescent lamp). A Spectralon panel (PSI Ltd., Drasov, Czech Republic) was used to calibrate the instrument. Because measurements were realized in laboratory conditions under controlled illumination, calibration was performed every time the device was turned on and regularly repeated after 10 measurements.

To allow the internal temperature to stabilize, both devices were turned on for at least 45 min before each measurement. The measurements made by the FS-4 device were used as reference data for the validation of the results obtained by the PP RP410 device.

### 2.3 VIs

VIs were calculated directly from the data measured by both spectroradiometers. Using the experimental data, a set of 41 previously published indices was analyzed (Table 1).

**Table 1:** The spectral reflectance indices analyzed in the study with the originally proposed scales of use

Index	Name	Calculation	Reference	Scale
MTCI	MERIS terrestrial chlorophyll index	(*R*_754_ − *R*_709_)/(*R*_709_ − *R*_681_)	[48]	Canopy
VOG1	Vogelmann index	*R*_740_/*R*_720_	[60]	Leaf
ZMI	Zarco-Tejada and miller index	*R*_750_/*R*_710_	[61]	Canopy
CI	Chlorophyll index	(*R*_750_ − *R*_705_)/(*R*_750_ + *R*_705_)	[62]	Leaf
NDRE	Normalized difference red-edge index	(*R*_790_ − *R*_720_)/(*R*_790_ + *R*_720_)	[63]	Canopy
SR705	Red-edge simple ratio	*R*_750_/*R*_705_	[64]	Canopy
REP	Red-edge position	700 + 40[*R*_670_ + *R*_780_)/2 − *R*_700_]/(*R*_740_ − *R*_700_)	[33]	Leaf
CVI	Chlorophyll vegetation index	(*R*_790_/*R*_550_)(*R*_680_/*R*_550_)	[65]	Canopy
LCI	Leaf chlorophyll index	(*R*_850_ − *R*_710_)/(*R*_850_ + *R*_680_)	[66]	Leaf
CI_GREEN_	Green chlorophyll index	(*R*_790_/*R*_550_) − 1	[67]	Canopy
GM1	Gitelson and Merzlyak index 1	*R*_750_/*R*_550_	[67]	Leaf
GNDVI	Green normalized difference vegetation index	(*R*_790_ − *R*_550_)/(*R*_790_ + *R*_550_)	[68]	Canopy
GM2	Gitelson and Merzlyak index 2	*R*_750_/*R*_700_	[67]	Leaf
Ctr2	Carter index 2	*R*_695_/*R*_760_	[69]	Leaf
TCARI	Transformed chlorophyll absorption ratio index	3 [(*R*_700_ − *R*_670_) − 0.2(*R*_700_ − *R*_550_)(*R*_700_/*R*_670_)]	[47]	Canopy
PPR	Plant pigment ratio	(*R*_550_ − *R*_450_)/(*R*_550_ + *R*_450_)	[70]	Canopy
BGI	Blue-green pigment index	*R*_450_/*R*_550_	[71]	Canopy/Leaf
SIPI	Structure-insensitive pigment index	(*R*_800_ − *R*_445_)/(*R*_800_ − *R*_680_)	[72]	Leaf
GI	Greenness index	*R*_554_/*R*_677_	[71]	Canopy
PRI	Photochemical reflectance index	(*R*_531_ − *R*_570_)/(*R*_531_ + *R*_570_)	[28]	Canopy
GVI	Greenness vegetation index	(*R*_682_ − *R*_553_)/(*R*_682_ + *R*_553_)	[73]	Canopy
Ctr1	Carter index 1	*R*_695_/*R*_420_	[69]	Leaf
MCARI	Modified chlorophyll absorption ratio index	[(*R*_700_ − *R*_670_) − 0.2(*R*_700_ − *R*_550_)] (*P*_700_/*R*_670_)	[45]	Canopy/Leaf
LIC2	Lichtenthaler index	*R*_440_/*R*_690_	[74]	Leaf
MTVI	Modified triangular vegetation index	1.5[1.2(*R*_712_ − *R*_550_) − 2.1(*R*_670_ − *R*_550_)]	[75]	Leaf
BN	Buschmann and Nagel index	*R*_790_ − *R*_550_	[76]	Leaf
NPCI	Normalized pigments chlorophyll ratio index	(*R*_680_ − *R*_430_)/(*R*_680_ + *R*_430_)	[35]	Canopy
mNDVI	Modified normalized difference vegetation index	(*R*_800_ − *R*_680_)/(*R*_800_ + *R*_680_ − 2*R*_445_)	[77]	Canopy
SR	Simple ratio	*R*_800_/*R*_680_	[78]	Canopy
RI	Red index	*R*_790_/*R*_680_ − 1	[67]	Canopy
EVI2	Enhanced vegetation index 2	2.5(*R*_790_ − *R*_680_)/(*R*_790_ + 6*R*_680_ − 7.5*R*_450_ + 1)	[79]	Canopy
NDVI	Normalized difference vegetation index	(*R*_790_ − *R*_680_)/(*R*_790_ + *R*_680_)	[80]	Canopy
PSNDa	Pigment-specific normalized difference	(*R*_790_ – *R*_680_)/(*R*_790_ + *R*_680_)	[25]	Leaf
RVI	Ratio vegetation index	*R*_790_/*R*_680_	[81]	Canopy
OSAVI	Optimized soil-adjusted vegetation index	(*R*_790_ − *R*_680_)/(*R*_790_ + *R*_680_ + 0.16)	[82]	Canopy
SPVI	Spectral polygon vegetation index	0.4 [3.7(*R*_800_ − *R*6_70_) − 1.2(*R*_530_ − *R*_670_)]	[65]	Canopy
RDVI	Renormalized difference vegetation index	*R*_780_ − *R*_670_)/((*R*_780_ + *R*_670_)^0.5^)	[75]	Canopy
MSAVI	Modified soil-adjusted vegetation index	[2*R*_790_ + 1 − [(2*R*_790_ + 1)^2^ – 8(*R*_790_ − *R*_680_)]^1/2^]/2	[83]	Canopy
EVI	Enhanced vegetation index	2.5(*R*_790_ − *R*_680_)/(*R*_790_ + 6*R*_680_ − 7.5*R*_450_ + 1)	[84]	Canopy
TVI	Triangular vegetation index	0.5 [120(*R*_750_ − *R*_550_) − 200(*R*_670_ − *R*_550_)]	[85]	Canopy
PSRI	Plant senescence reflectance index	(*R*_680_ − *R*_500_)/*R*_750_	[35]	Leaf
RVSI	Red-edge vegetation stress index	[(*R*_712_ + *R*_752_)/2] – *R*_732_	[86]	Canopy
NPQI	Normalized phaeophytinization index	(*R*_415_ − *R*_435_)/(*R*_415_ + *R*_435_)	[87]	Leaf

To assess their interrelationship, we selected indices developed for broadband sensors using visible and near-infrared wavebands, indices using narrow red-edge bands, or other specific properties. VIs used included normalized difference indices (e.g., NDVI), simple ratio indices (e.g., SR705), triangular VIs (e.g., TVI), modified versions of these three types of indices (e.g., MTVI), and indices based on red edge position (e.g., REP). The closest wavebands for each instrument were used for index calculation. For indices where the values in the NIR band exceeded the range measured by the PP RP410 device, the NIR values in the available band (790 nm) were used, which already ensured the plateau values reached in the NIR band.

**Figure 1:**
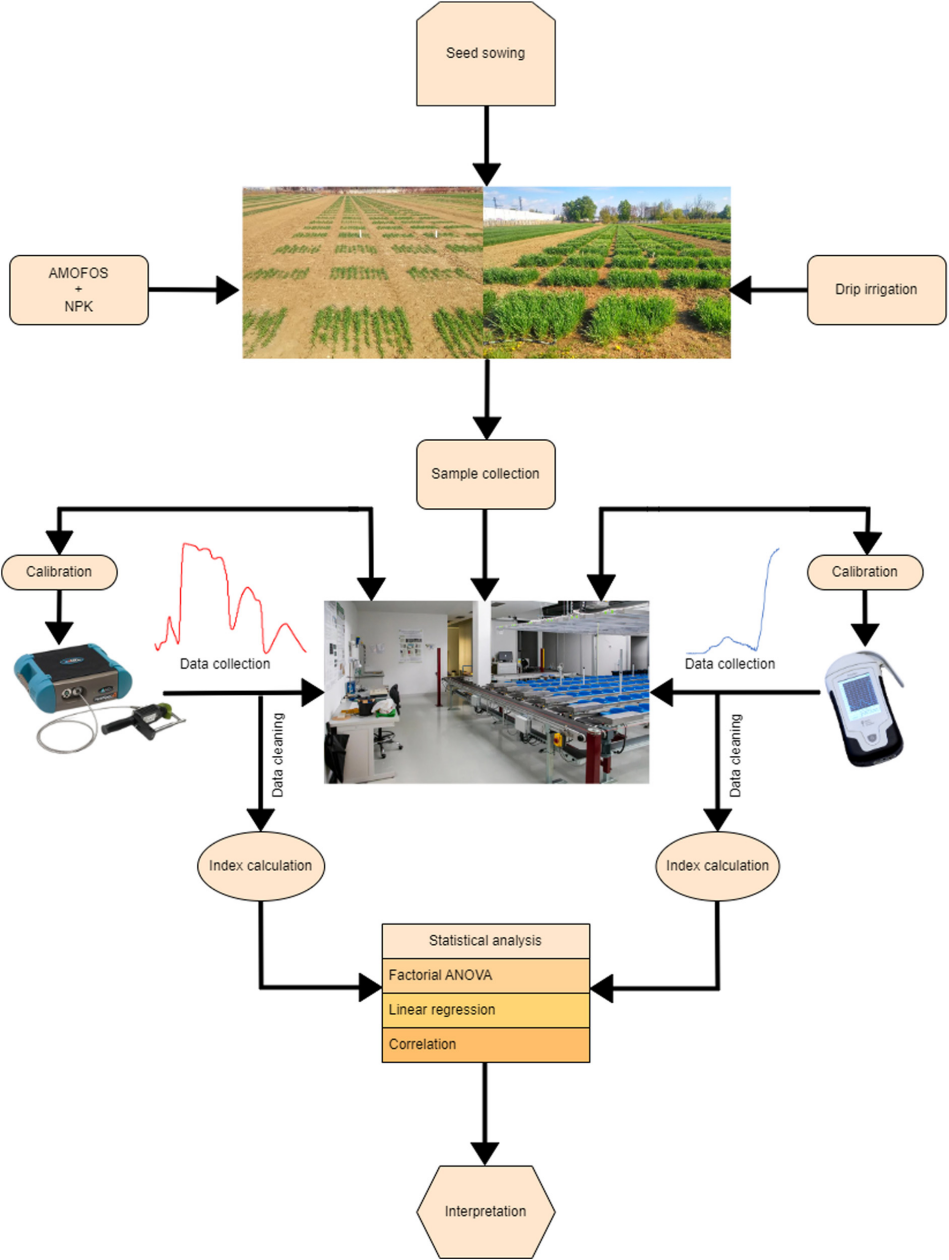
Schematic overview of the experimental setup, activities, and analyses applied in the study.

The scheme of the overall workflow of the experiments and analysis presented in this study is shown in Figure 1.

### 2.4 Statistical analysis

The datasets of the parameters calculated for each leaf sample were processed and analyzed using analysis of variance (ANOVA) and Duncan’s test using (STATISTICA 10, StatSoft, Tulsa, USA). Two-factor ANOVA was applied to analyze differences among a set of genotypes and individual measuring dates. The same software was also used to perform the correlation analyses and calculate the Pearson correlation index for each correlation.

The size of the effects for individual assessed factors (genotype, date) was estimated from the partial eta-squared (*η*_p_^2^) values produced by ANOVA [88]. The partial eta-squared values are defined as the proportion of variance associated with each of the factors in an ANOVA analysis [89]. As the measurements using two devices were performed using the same number of measurements within the same statistical design, the partial eta square values for individual parameters can be compared.

## 3 Results and discussion

This study compared a relatively new portable sensor with an existing scientific standard (control) sensor for in-field use. The PP RP410 has been successfully used in several studies [90–93], demonstrating its usability. So far, however, no studies have been conducted that would directly demonstrate its usability in measurements of the spectral reflectance of leaves in the maximum range of wavelengths (380–790 nm), which the UVIS version of the detector allows for evaluating.

**Figure 2:**
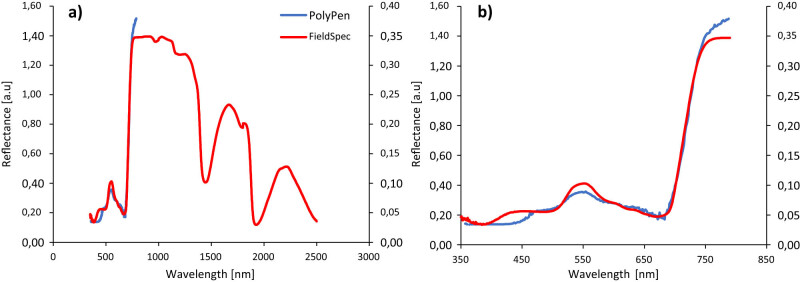
Reflectance spectra of two tested devices in the entire range of their measuring range (a) and in the range VNIR, used for calculation of indices (b). On the primary axis, the blue curve obtained by the PP instrument is shown, and the red curve obtained by the FS-4 is shown on the secondary axis. Both records were obtained on an identical sample.

The FS-4 is a high-resolution device that covers wavelengths between 350 and 2,500 nm, whereas the PP instrument with a UV/VIS detector version can only cover the spectral band in the 380–790 nm range. Specifically, this spectral band was used for VI calculation and correlation analysis (Figure 2b). Figure 2a shows the reflectance spectra (curves) of both devices in their entire measurement range.

The spectral reflectance curves obtained by both devices (Figure 2) indicate that the curve obtained by the FS-4 device is much smoother than the curve obtained by the PP device. A next comparison of the spectral curves obtained in the VNIR region by the two spectroradiometers revealed minor variations between the curves, with the blue band indicating the first difference when the reflectance curve obtained by the PP device begins to increase even at higher wavelengths (∼480 nm), while the curve obtained by the FS-4 device shows a more typical pattern for healthy wheat leaves [94,95] and increases already in the region of ∼410 nm, previous studies [61] indicated that the reflectance in the blue region is mainly affected by the content of carotenoids, although chlorophyll also plays a role. Due to chlorophyll’s intense and overlapping absorption, spectral characteristics of carotenoids become less apparent as the amount of chlorophyll in leaves increases [96]. The differences between the curves were also manifested in the area of the green peak (550 nm), where we note a higher reflectance measured with the FS-4 device compared to the PP device. Antenna pigments and the reaction centers involved in photosynthesis are excited by both blue and red radiation. However, chlorophyll does not effectively absorb the majority of green light, resulting in minor maxima in reflectance and transmittance near 550 nm [97]. The green peak of the green band (540–560 nm), in addition to the red edge band (680–750 nm), is used to estimate the amount of chlorophyll in leaves [61]; therefore, when utilizing the PP device, these discrepancies may result in an underestimating of the chlorophyll content when using a wavelength of the green peak for calculation of VIs. On the other hand, however, the area of the red edge is characterized by an almost complete overlap of both curves. This finding assumes that the most significant agreement between the outputs of both devices should be in this area.

**Figure 3:**
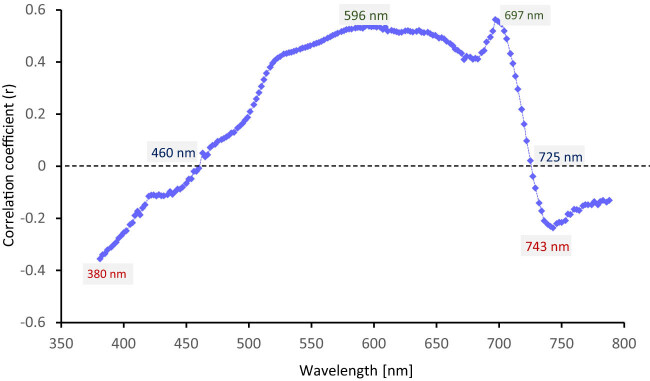
Correlogram of wavelengths in the range 380–790 nm. The significant wavelengths are identified (with maximum, minimum, or zero *r*).

In the next step, we compared the spectral data from the maximum wavelength in which the PP device operates with the spectral data obtained by the FS-4 device from the same wavelength segment (380–790 nm) to determine the correlation coefficients of individual wavelengths which will help us to better reveal the match in specific spectral bands (Figure 3).

In terms of correlation, we noticed a growing inverse correlation trend at the beginning of the curve in the region of 380 nm (UV), which turns positive in the blue region with a very low correlation (*r* = 0.05) between devices at the beginning of the blue region (463 nm), but at the end of it (520 nm) the correlation rate is already higher (*r* = 0.40). At the beginning of the green region (530 nm), we can observe an increasing correlation trend (*r* = 0.41) with a progressive increase towards higher wavelengths, and at the end of it (600 nm), the correlation is even higher (*r* = 0.54). In the region of the red band between wavelengths 620–680 nm, we noticed a dip in the correlation curve, which was manifested by a decrease in the correlation from *r* = 0.51 to *r* = 0.41; however, the subsequent increase up to the highest peak of the correlation curve (697 nm; *r* = 0.56) indicates that the very beginning of the red edge region is the region where both instruments correlate the most. From the peak of the correlation curve, however, we notice a sharp decline in the correlation in the region of the red edge up to the wavelength of 726 nm, where the correlation rate is almost zero, turns negative, reaches a negative peak (746 nm), and continues in a downward negative trend up to the plateau of 790 nm.

The wavelengths obtained by both spectroradiometers were used to calculate 41 VIs directly. We chose indices using visible and near-infrared wavebands, indices utilizing restricted red-edge bands, or other particular attributes to evaluate their interdependence. Some indices intended to be utilized only at the canopy scale have also been incorporated here when relevant findings were anticipated or to examine how they fared at the leaf scale.

**Figure 4:**
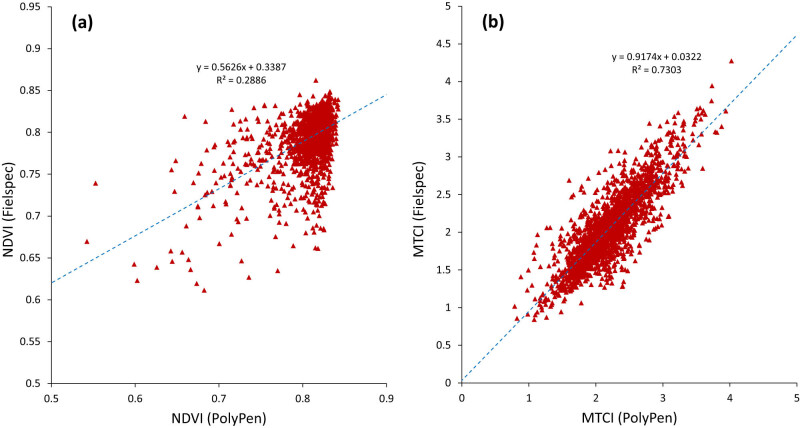
Examples of representative indices showing different levels of correlations between the values ​​obtained by the two devices: (a) NDVI and (b) MTCI. Points represent average reflectance data of leaves of individual wheat genotypes. Data were fitted to a linear function. The graph contains individual measurements of more than 1700 leaves from all observed genotypes and all measuring dates. Paired data points from measurements captured by PP and FS-4 devices were always performed on the same leaf.

In Figure 4, we present two representatives of indices showing different (and contrasting) levels of correlations between the values obtained by the two devices. There seems to be more spread in the mid-range NDVI values, especially around 0.65 to 0.75. At high NDVI values, the data points show a typical saturation effect, when variations in green biomass are no longer reflected in the NDVI [98]. On the other hand, in the case of MTCI, the point distribution shows a better consistency of measurements between the two instruments with less variability than we observed in the NDVI data, which is known for the variability of values obtained by different devices [49].

**Figure 5:**
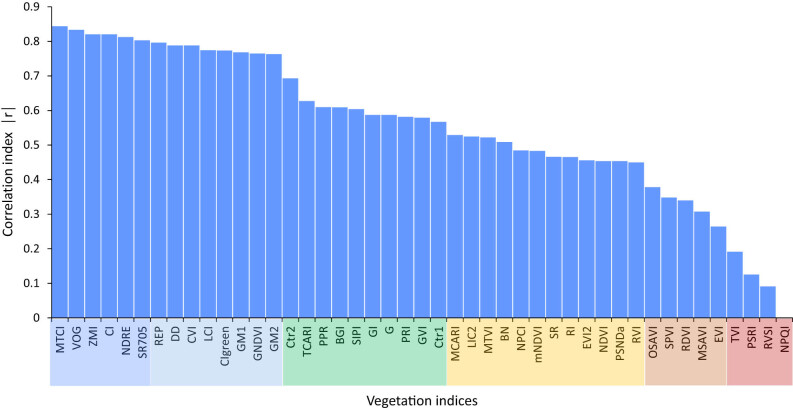
Absolute values of correlation indices derived from the correlation analyses of individual VIs calculated from spectral data of PP and FieldSpec spectroradiometers. The full name of every vegetation index is shown in Table 1.

The numerical values of correlation coefficients (their absolute values) for 41 VIs (Figure 5) clearly show the grouping of individual parameters based on the correlation level but also based on the spectral bands used for calculations. It is clear that the VIs, including the red-edge band, usually perform better than VIs that use visible bands. The highest-ranked VIs use reflectance at wavelengths of the red edge, a sharp transition from low reflectance at an inflection point of around 680 nm to high reflectance at around 750 nm. These include indices such as MTCI, ZMI, or VOG. Indices using a combination of the green band and the red edge band (CI_green_, GM1, GNDVI) also showed a very good level of correlation between devices. Replacing the red band with green [68] in the case of GNDVI resulted in a significantly higher agreement between the outputs of both devices, compared to the conventional NDVI, which was ranked almost among the last. A surprisingly high degree of association between devices was achieved by the blue-green pigment index, and plant pigment ratio using the blue band of carotenoid absorption with a wavelength of 450 nm, in which the agreement between devices was very weak (Figure 3). It seems, that the use of wavelengths of the green band centered at 550 nm, in which the devices showed a good level of correlation, managed to correct the differences caused by the use of the wavelength of 450 nm. The weakest agreement between devices was shown by the NPQI index, which uses only even lower blue band wavelengths for calculation.

Comparing the indices between these two devices is therefore possible, but it depends on which part of the spectrum the index is calculated from.

**Figure 6:**
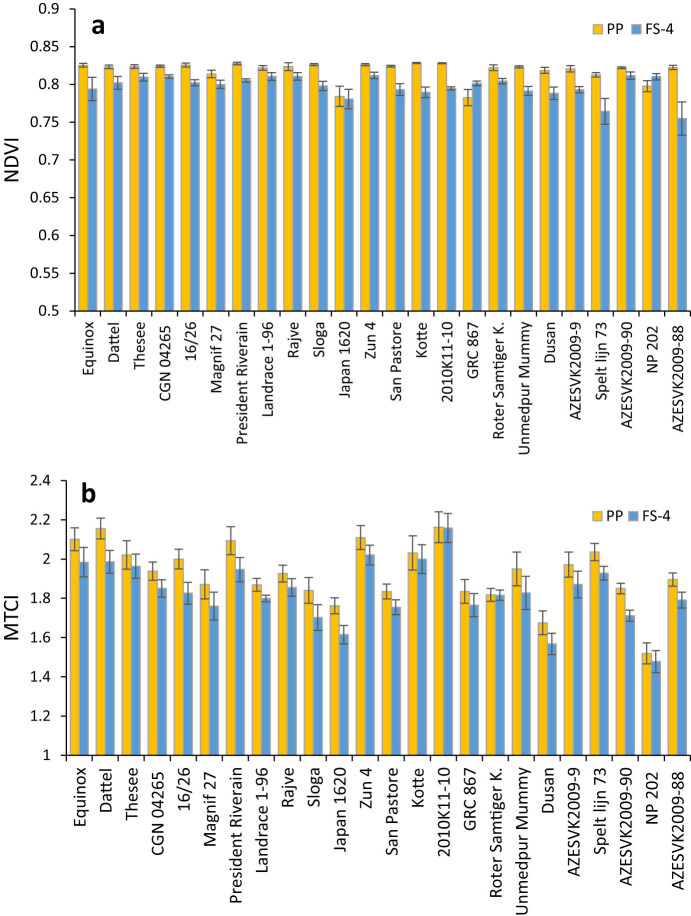
Example of the values of two parameters differing in correlation level found in individual wheat genotypes in a single measuring date: (a) NDVI and (b) MTCI. The pairs of values represent the parameters calculated from the records of PP (column left) and FS-4 (column right).

It is well known that the physiology, morphology, or anatomy of a plant species affects its spectral characteristics [99,100]. The optical properties of leaves (i.e., leaf reflectance, transmittance, and absorbance), which vary not just across species but also within species, are a result of variations in the biochemistry and internal structure of the leaves [18,101] and it is, therefore, logical to expect the different performance of VI for distinct wheat genotypes. To better understand how the indexes can react differently with different genotypes, we selected the conventional NDVI index and the index that showed the highest agreement between the devices: MTCI. Figure 6 shows the response of selected indices in relationship with distinct wheat genotypes.

It is clear that NDVI showed a much lower genotypic variability, as observed in measurements using both devices. Some inconsistency between the values can be attributed to different areas from which the spectral reflectance is captured. While the PP has a tiny screen and most of the measurements were performed in the middle of the leaf blade, the FS-4 takes a larger area and better reflects also the leaf margins. The effects of stress or aging occur first in leaf margins; therefore, spotting the central leaf position only may lead to more stable values (as observed in NDVI measured by PP). The ability of NDVI to distinguish genotypic variability could be influenced by the growth stage of wheat genotypes due to the fact that grain-filling stages (Z71–79) appeared the most valuable for detecting NDVI-related genetic differences in wheat genotypes [102] while our plants were measured in Z39–47 stage which could be the cause of the detected negligible genotypic variability.

On the other hand, the genotype differences are very clearly demonstrated in the case of parameter MTCI. In this parameter, genotypic differences were very high, and the effects of the screen size were not so evident.

**Figure 7:**
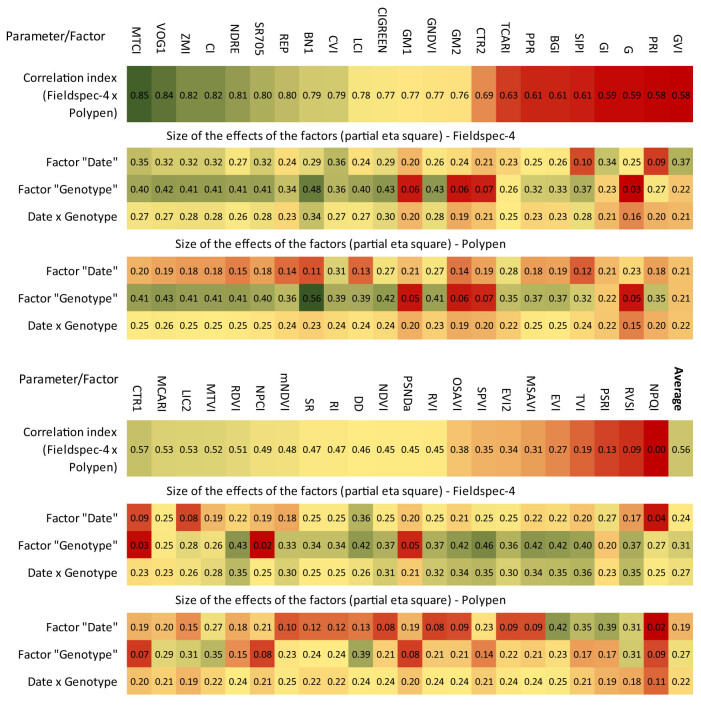
A heatmap showing a comparison of the effect size of the two factors (genotype, date) and their interactions assessed by ANOVA test using the partial eta-squared (*η*_p_^2^) values. Separately are presented effect sizes for the FS-4 and PP record-based parameters. Individual parameters are ordered from highest to lowest based on their correlation indices obtained by analysis of the relationship between the values obtained by the FS-4 device and the PP device. The green color represents the favorable (higher) values of the statistical indicators, the yellow color indicates the moderate level, and the red color indicates the low values of the indicators.

This observation suggests that the parameters showing a higher effect of the main factors might be those with a higher level of correlation between the two devices. Since the measurements were carried out during the entire growing season, we decided to test not only the influence of the “Genotype” factor on the performance of all indices but also the influence of the “Date” factor and their mutual interaction. To evaluate the effect size of the factor (“date,” “genotype” and their interaction), we used the partial eta-squared (*η*_p_^2^) for the two-way ANOVA (Figure 7).

It is evident from the results that the indices calculated from the spectral data of the FS-4 instrument were generally better able to distinguish the influence of the factor “date” compared to the indices obtained from the spectral data of the PP, which ultimately makes them more sensitive to changes caused by the aging of leaves during the growing season.

The first ten best-correlated indices demonstrated a relatively balanced ability to distinguish not only the influence of individual factors but also the interaction of factors on their performance. An interesting finding was that other indices with high values of correlation coefficients (GM1, GM2, CTR2, G) were almost unable to distinguish genetic variability, which was manifested by very low values of *η*_p_^2^ for the factor “genotype,” but also for the factor “date” and their mutual interaction. In the case of such a wide collection of genotypes, using only these indices would, therefore, not be appropriate. In this regard, BN1 proved to be the best because it significantly dominated the capacity to distinguish intraspecies variability among all indices.

Different types of spectrometers, or even models made by the same company, may have drastically different spectral performance and model accuracy [103]. Consequently, it is possible to anticipate variations in spectral reflectance data when comparing two different sensors. The stability, signal-to-noise (SNR), configuration, illumination, detector performance, fore-optic and fiber optic properties, stray light contribution, and warm-up time of the spectrometer are all important factors [104]. Since the instrument operator has no control over internal parameters (such as detector performance, calibration quality, dispersion element and stability, and stray light), all other potential external elements must be maintained constant [105,106].

Although there is a clear correlation between device temperature and sensor readings in field conditions [107], the effect of temperature on the performance of the optical sensors was probably negligible since the measurement with each device was performed in the laboratory conditions and thus at a stable temperature, which at the same time also eliminated temperature oscillations that could lead to instrument modifications as a result of thermal expansion [108]. Also, before beginning data collection, both instruments had a proper time to warm up or reach temperature equilibrium with the environment [109]. The spectral resolution, spectral range, and SNR ratio are found to have a direct impact on the estimation accuracy of sensors [110]. Therefore, we have reason to believe that one of the possible reasons for the differences between the optical sensors may be the different spectral resolution of the devices, which is a measure of the narrowest spectral feature that the device can distinguish [111]. FS-4 has a spectral resolution of 3 nm, while FS-4 has a spectral resolution of 8 nm. Therefore, the PP would not be able to identify small spectral characteristics captured by the FS-4 device.

PP and FS-4 with contact probes can be considered active sensors that assess the light reflectance of plants, soil, or other surfaces using light released in one or more specified wavebands by the sensor, not ambient light. The selection of a light source (e.g., Xenon, Halogen, or LED) is therefore a very important factor. The combination of optical filters and source of light is an important factor for both the detected light and the emitted wavebands [107]. PP uses a xenon lamp as a light source, which produces a fairly flat visible spectrum but is more unstable than a halogen bulb [112], which is implemented in the contact probe of FS-4. According to Schuerger and Richards [113], using a halogen lamp as a light source produces smooth and easy-to-understand spectra of healthy and stressed plant canopies in the entire range of reflectance spectra (350–2,500 nm). Halogen lamps, however, have a poor blue emission, which may have led to a weak agreement between the outputs of both devices in a blue region. Using such a light source can also have the drawback of producing a significant temperature signature and the possibility of the leaf wilting after prolonged exposure. Another potential cause of the differences (even though it was not quantified) could be the SNR which can vary between devices. Even though each of the aforementioned potential sources of inaccuracy seems insignificant, they could have contributed to the slight but noticeable discrepancies found in the same-leaf spectra obtained from the various spectrometers [111].

## 4 Conclusions

Based on whole-season records of spectral reflectance indices obtained from two spectroradiometers on a wide range of wheat genotypes, the comparison of the accuracy of records of low-cost multispectral leaf sensor PP with a standard, highly sensitive hyperspectral device (FS-4) was performed. We noted that spectral reflectance curves obtained by the PP differed slightly from those obtained by the hyperspectral sensor with high resolution on the same samples. Correlation analysis identified parts of the curve that correlate very well with each other, but also segments of the curve (mainly in the blue area) with low correlation. Correlation analyses of 41 spectral reflectance indices, which can be calculated using both devices, showed the best degree of correlation and, therefore, the highest agreement between the outputs of the multispectral and hyperspectral sensor achieved indices using green and red-edge bands. The highest-ranked VIs use reflectance at wavelengths of the red edge, a sharp transition from low reflectance at an inflection point of around 680 nm to high reflectance at around 750 nm. These include indices such as MTCI, ZMI, or VOG. Indices using a combination of the green band and the red edge band (CIgreen, GM1, GNDVI) also showed significant variation among genotypes and a very good level of correlation between devices. On the contrary, we also identified a group of indices with low correlation, including several frequently used parameters, such as NDVI. One of the possible reasons is a low variation in their values in plants with a good nutrition level despite varietal differences in leaf properties. The FS-4 records showed a higher genotypic variability, which can also be attributed to a larger area screened and better coverage of spatial heterogeneity compared to PP, with a very low screened area, where the measurements were taken mostly in the center of the leaf blade. That assumption was also supported by the analyses of the effect size of the main factors, which were, on average, higher in the FS-4 device than in PP. That also led to a lower ability to distinguish seasonal changes using a low-cost multispectral device. However, there were also parameters with an acceptable level of sensitivity and a good level of mutual correlation between the records of the two devices.

Based on the results, it can be concluded that for assessment of the genotypic and seasonal variability of the leaf traits using the multispectral records of the PP instrument, the selection of appropriate parameters is crucial. The study enabled to identify a set of parameters able to recognize specific patterns of individual genotypes, which were consistent with the records obtained by a highly sensitive hyperspectral analyzer. Thus, the efficient and reliable use of the PP device in the assessment of wheat genetic resources is possible, but only if appropriate parameters are selected. Further analyses to associate the specific parameters to particulate leaf traits of the genotypes will be needed.
